# Supplementary material for: Patterns of Intron Gain and Loss in Fungi
Source: PLoS Biol. 2004 Nov 30;2(12):e422. doi: 10.1371/journal.pbio.0020422 (PMC532390; doi:10.1371/journal.pbio.0020422)
Supplement: Table S1 — Also available at http://genes.mit.edu/NielsenEtAl/. (4.3 MB ZIP). [file pbio.0020422.st001.zip › NielsenEtAl/html/246.html]

AN1662.1.NCU09442.1.MG04898.1.FG07515.1


```
 CLUSTAL W (1.82) Multiple Sequence Alignments - Introns Inserted


Sequence 1: NCU09442.1	170 aa
Sequence 2: FG07515.1	163 aa
Sequence 3: AN1662.1	177 aa
Sequence 4: MG04898.1	198 aa
Alignment Length: 235 aa
Number Identitical Residues: 73 aa
Alignment Score (without introns) 3664


MG04898.1 	MVQQFGPEPPPGLRKADAGFDAENAPNHEG0IEMAFAEKG1SIGPSAGQARCQTWGGASV
NCU09442.1	----MS-----VTTQVPKDFDAENADNFED0IEKQFAVK-~-------------------
FG07515.1 	----MDPLDNMAGAPVPKNFDAENAQNNED0IEKQFAVK-~-------------------
AN1662.1  	---------------MSKPFDPEQAQNLED0MEKQFAVK-~-------------------
          	                   **.*:* * *. :*  ** *                     

MG04898.1 	RGTRLMDGNLAV~IQHMETYWKILKNKRGSQLRLTKIDDEIYEHLKKDFPELDPAVDIDE
NCU09442.1	-----------A~VQQMATYWAILEKVPGSKLRLTKMDDDIFEHLKRDFPEFDPAEPINE
FG07515.1 	-----------V~VQHMQTYWSILERVKGSSLRLTKIDDEIMEHLKEAFPDFDPAAKVDE
AN1662.1  	-----------A1VEHLMTYWSILEKVPGSQLRLTKIDDEIYEHFKAEFPDFDPAETIDE
          	           . :::: *** **:.  **.*****:**:* **:*  **::***  ::*

MG04898.1 	DVMKNKENKERWRKFLNFYDKKVEDFNFGTMLRKNPKDDVEVENRIIFV1P---------
NCU09442.1	DEMKSKTGKERWRTFMMAYDKKIDDYNFGTLLRASPKTEYGEKE-TIFV~P---------
FG07515.1 	DAMKSKDGKNRWREFMMKYEKKVDDYNFGTMVRDSPKVEYEENT-TIFV~P---------
AN1662.1  	DKMKSKEGKEKWRNWINQYEKKIHDFNFGTMLRANPKTEYERDN-TIFG~ESSEALELLN
          	* **.* .*::** ::  *:**:.*:****::* .** :   .   **   ::.: .  .

MG04898.1 	---------------RMQFYAFEIAR2NKAGLNDWIYEQEHPEEKKE-------------
NCU09442.1	---------------RMQFYAVEIAR2NRAGLNDWIYEEAEKERQKAKGNTQGSSSSSSA
FG07515.1 	---------------RMQFYAFEIAR2NRAGLNDWIYEQAQAKKAKSK------------
AN1662.1  	MDWDVWLTGFLLLAMRMQFYAIEIAR2NRAGLNDWVYEQAQARAKNSSS-----------
          	 . .   :.    : ******.**** *:******:**: . .  : ..           

NCU09442.1	
FG07515.1 	
AN1662.1  	
MG04898.1 	
          	
```
